# Excess Dally-like Induces Malformation of *Drosophila* Legs

**DOI:** 10.3390/cells13141199

**Published:** 2024-07-15

**Authors:** Xubo Zhang, Yi Wang, Wenting Zhao, Shumin Yang, Bernard Moussian, Zhangwu Zhao, Jianzhen Zhang, Wei Dong

**Affiliations:** 1 Shanxi Key Laboratory of Nucleic Acid Biopesticides, Research Institute of Applied Biology, Shanxi University, Taiyuan 030006, China; 2 Institut National de Recherche pour l’Agriculture, l’Alimentation et l’Environnement, Centre National de la Recherche Scientifique, Institut Sophia Agrobiotech, Sophia Antipolis, Université Côte d′Azur, 06108 Nice, France

**Keywords:** Dlp, malformated legs, Dll, cell apoptosis, Wg

## Abstract

Glypicans are closely associated with organ development and tumorigenesis in animals. Dally-like (Dlp), a membrane-bound glypican, plays pivotal roles in various biological processes in *Drosophila*. In this study, we observed that an excess of Dlp led to the malformation of legs, particularly affecting the distal part. Accordingly, the leg disc was shrunken and frequently exhibited aberrant morphology. In addition, elevated Dlp levels induced ectopic cell death with no apparent cell proliferation changes. Furthermore, Dlp overexpression in the posterior compartment significantly altered Wingless (Wg) distribution. We observed a marked expansion of Wg distribution within the posterior compartment, accompanied by a corresponding decrease in the anterior compartment. It appears that excess Dlp guides Wg to diffuse to cells with higher Dlp levels. In addition, the *distal-less* (*dll*) gene, which is crucial for leg patterning, was up-regulated significantly. Notably, *dachshund* (*dac*) and *homothorax* (*hth*) expression, also essential for leg patterning and development, only appeared to be negligibly affected. Based on these findings, we speculate that excess Dlp may contribute to malformations of the distal leg region of *Drosophila*, possibly through its influence on Wg distribution, *dll* expression and induced cell death. Our research advances the understanding of Dlp function in *Drosophila* leg development.

## 1. Introduction

The regulation of organ development is a highly intricate process that involves the orchestration of multiple signaling pathways. The subtle interplay among these pathways is critical for organ size determination and pattern formation. Heparan sulfate proteoglycans (HSPGs) have emerged as key regulators of these signaling cascades [1,2]. HSPGs are a group of macromolecules present on the cell surface and in the extracellular matrix [3,4,5]. They are comprised of a core protein and covalently attached heparan sulfate glycosaminoglycan (GAG) chains [6,7]. HSPGs play a pivotal role in cell signaling and morphogen gradient formation [8]. They serve as co-receptors, modulating the threshold and duration of numerous signal transductions [9].

Among the HSPGs, glypicans are cell surface proteoglycans bound to the cell membrane via a glycosylphosphatidylinositol (GPI) anchor [10]. In vertebrates, six glypicans are recognized [11], while in *Drosophila* there are two types of glypicans [12], Dally and Dlp. Of these, Dally is associated with GPC3 and GPC5, while Dlp corresponds to GPC1, GPC2, GPC4 and GPC6. These unique glypican molecules do not possess transmembrane domains [13]. Instead, they attach to cell surfaces through GPI anchors, facilitating interactions with secreted ligands. Dally and Dlp are implicated in regulating the concentration gradient formation of morphogens, including Wg and Hedgehog (Hh) [14,15,16]. Dlp has opposite regulating effects on tissues expressing low and high levels of Wingless. Dlp reduces high-level Wg activity but enhances low-level Wg signaling [17]. In addition, Dlp is also involved in Hh reception by acting with the Hh receptor Patched (Ptc) [18]. Dlp also mediates the feedback control of the interdependence between Hh and Wnt signaling during GSC (Germline Stem Cell) progeny differentiation, highlighting its crucial role in coordinating the crosstalk between these signaling pathways [19].

The *Drosophila* leg provides a good model for investigating the molecular and cellular mechanisms of signaling and their outcome during organogenesis. Legs arise from specific ectodermal cells that are genetically specified during embryogenesis, giving rise to imaginal discs which eventually develop into adult appendages [20,21]. The adult leg consists of distinct segments along the proximal–distal (P–D) axis, including the coxa, trochanter, femur, tibia and five tarsal segments [22]. Establishing proper subdomains within the leg disc is critical for the formation of the corresponding well-structured segments in the adult leg. The correct patterning of the leg disc is tightly organized by the three morphgens Hh, Wg and decapentaplegic (Dpp). Hh is expressed in the posterior compartment of the leg disc and diffuses to the anterior compartment as a short-range signal [23]. It directly activates the expression of Wg and Dpp. Dpp is expressed in a stripe abutting the anterior–posterior compartment boundary, with higher expression in the dorsal region and lower expression in the ventral region [24]. Wg is expressed in a wedge-shaped pattern in the ventral–anterior region [25]. Both Wg and Dpp are required for defining the target genes’ spatial domains along the P–D axis and D–V axis [21]. Interestingly, Wg and Dpp act antagonistically to provide positional information for the establishment of the D–V axis, while they act cooperatively to direct the P–D axis formation [26,27]. 

Homothorax (Hth), Distal-less (Dll) and Dachshund (Dac) are the vital target genes involved in leg formation [23]. The early leg disc is patterned by the opposing activities of Homothorax (Hth) and Distal-less (Dll) along the P–D axis. These two factors define the proximal and distal domains of the leg, respectively [26]. Dachshund (Dac) is activated later in an intermediate domain of the leg disc, which corresponds to the development of medial adult leg segment formation including the first tarsal segment, the tibia and the presumptive femur [28]. During the mid-third larval instar, the expression domains of Dll and Dac overlap in a specific region. Both genes are induced by the combined activities of the Wg and Dpp signaling pathways. However, Dll and Dac are further regulated by Brk, which acts as a repressor for both [28]. Dpp and Wg signaling act antagonistically to repress Hth expression. This combined repression restricts Hth localization to the most proximal region of the leg disc [26].

In this study, we found that overexpression of Dlp in *Drosophila* legs led to a malformation of legs. Excess Dlp results in obvious cell death without apparent cell proliferation alteration. Additionally, Wg distribution was aberrant, and the expression of its downstream gene *dll* was also mis-regulated. Our study provides valuable insight into the role of Dlp in the development of *Drosophila* legs.

## 2. Materials and Methods

### 2.1. Drosophila Stocks

Flies were cultured on standard cornmeal food. The stocks used in this study were UAS-*dlp* (Bl#9160), *wg*-lacZ (Bl#11205) and UAS-*p35* (Bl#5072), and they were purchased from BDSC. UAS-*dlp*-RNAi (VDRC#10299) was obtained from VDRC. The UAS-GFP, *hth*-lacZ, *dll*-lacZ, *tub*-Gal80^ts^ and *en*-Gal4 fly lines were obtained from Prof. Jie Shen.

### 2.2. Immunochemistry Staining

The leg discs of 3rd instar larvae were dissected and fixed in a 4% formaldehyde (Damao Chemical Reagent Factory, Tianjin, China) solution for 40 min. The samples were rinsed with PBT 4 times and shaken in PBT for 1 h on a rocking shaker. Next, samples were incubated with the primary antibody at 4 °C overnight. Then, they were rinsed with PBT four times and kept in PBT for another 30 min before incubation with the secondary antibody for 1 h at room temperature. Samples were rinsed with PBT four times and shaken in fresh PBT for another 1 h. The following primary antibodies were used in this study: mouse anti-Dlp (1:20; 13G8, Developmental Studies Hybridoma Bank, Iowa City, IA, USA), mouse anti-β-Gal (1:200; Promega, Madison, WI, USA, Z3788), mouse anti-Wg (1:100; 4D4, Developmental Studies Hybridoma Bank, Iowa City, IA, USA), rabbit anti-Caspase3 (1:100; Asp175, Cell Signaling Technology, Danvers, MA, USA), rabbit anti-PH3 (1:500; pSer10, Cell Signaling) and Anti-Dac (1:50; 13G8, Developmental Studies Hybridoma Bank, Iowa City, IA, USA). Secondary antibodies were goat anti-mouse DyLight 549 (1:200; Agrisera, Vännäs, Sweden) and goat anti-rabbit DyLight 549 (1:200; Agrisera). The leg discs were mounted on glass slides, and images were collected by an inverted fluorescence microscope (EVOS FL, Life Technologies, Carlsbad, CA, USA) or a confocal laser scanning microscope (LSM 880, Carl Zeiss, Jena, Germany).

### 2.3. Measurements and Data Statistical Analysis

To avoid the lethal effect of Dlp mis-expression at the early developmental stage, Gal4 driver was combined with *tub*-Gal80^ts^. The F1 generation was incubated at 18 °C before the second instar. Then, the larvae were transferred to 30 °C to induce the overexpression of *Dlp* or RNAi against *Dlp* for 2 or 3 days. Subsequently, the flies were shifted to 18 °C to reach the adult stage. Adult flies or legs were dissected and imaged using a Multifocus Imaging System of a microscope (MV PLAPO 1X, Olympus, Tokyo, Japan). Images were analyzed by Fiji software, version 2.15.1.

For the statistical analysis of the distal part of adult legs, we measured the tarsal segment lengths of hindlegs. The results were analyzed by one-way ANOVA with Tukey’s test. For the quantification of cell proliferation in leg discs, PH3-positive puncta density within the P compartment was calculated by determining the ratio of positive puncta to the P compartment size. Similarly, for Caspase-3 staining, the number of positive puncta was counted in both the P compartment and the central region of the leg disc. For the quantification of Wg distribution and *wg*-lacZ domains, the size of the apparent locations was measured by Fiji software. Quantification of *dll* expression was achieved by measuring the fluorescence intensity of the apparent increased *dll*-lacZ expression regions in the P compartment and adjacent areas. A histogram was plotted and statistical analysis was conducted using GraphPad Prism 8.0. All two-mean comparisons were created using Student’s *t*-test.

## 3. Results

### 3.1. Extra Dlp Expression Induces Leg Deformities

**Figure 1:**
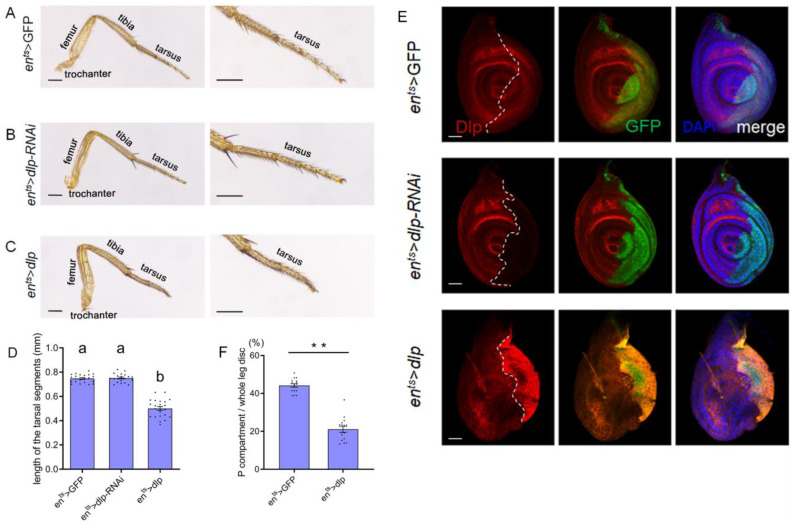
Overexpression but not repression of Dlp-induced defects of *Drosophila* legs. (**A**). Normal adult leg segmentation in control *en^ts^* > GFP flies. (**B**). Adult legs of *en^ts^* > *dlp*-RNAi flies show no visible defects. (**C**). Overexpression of Dlp in the *en^ts^* > *dlp* leg disc results in a characteristic shrinkage of the distal portion of the adult leg. The scale bar is 0.3 mm. (**D**). Statistical analysis of distal part length of adult legs shows the tarsal segments of adult legs are abbreviated significantly in *en^ts^* > *dlp* flies, while they are normal in *en^ts^* > *dlp*-RNAi flies (mean ± SEM; *en^ts^* > GFP, *n* = 22; *en^ts^*> *dlp*-RNAi *n* = 20; *en^ts^* > *dlp*, *n* = 23). Bars with different letters indicate significant statistical differences between the groups (*p* < 0.01). (**E**). Anti-Dlp staining (red) shows that Dlp is roughly uniform in the control *en^ts^* > GFP leg discs; Dlp is effectively repressed in the P compartment of *en^ts^* > *dlp*-RNAi leg discs; Dlp is up-regulated in *en^ts^* > *dlp* leg discs. The scale bar is 50 μm. (**F**). Statistical analysis of the proportion of the P compartment area to the whole leg disc (mean ± SEM; *en^ts^* > GFP, *n* = 16; *en^ts^* > *dlp*, *n* = 16). The white dashed lines indicate the A–P compartment boundary. Asterisks indicate significant differences (*p* < 0.01).

Dlp regulates multiple aspects of biological processes, including signal trafficking and morphogen transport. Dysregulation of Dlp expression may lead to abnormal organ development. To investigate the impact of *dlp* expression levels on leg development, we manipulated Dlp levels in *Drosophila* legs using the Gal4/UAS system. Under the control of the *en*-Gal4 driver, which is expressed in the posterior compartment of the leg disc [28], we modulated the expression levels of *dlp* within this region. To avoid the lethal effect of abnormal *dlp* expression, we applied a *tub*-Gal80^ts^ to manipulate the Gal4 activity. At 18° C, the Gal4 activity was repressed, while it was de-repressed at a high temperature (30 °C). The 2nd-instar larvae with *tub*-Gal80^ts^, *en* > dlp (*en^ts^* > *dlp*) were cultured at 30 °C for 3 days and then transferred to 18 °C until the adult stage. This resulted in consistent malformation of legs (Figure 1C). Compared to normal legs (Figure 1A), the tarsal segments were abbreviated and fused significantly (Figure 1D), while other segments still appeared normal (Figure 1C). Occasionally, some legs were misplaced or totally missing (Figure S1). Conversely, we found that repression of *dlp* showed few signs of leg deformities (Figure 1B) and no mis-location phenotype. This suggests that up-regulation rather than down-regulation of *dlp* induces malformed or mis-located legs.

To validate the manipulation of the Dlp expression, we conducted anti-Dlp staining to assess Dlp levels. The analysis revealed an up-regulation of Dlp expression in *en^ts^* > *dlp* leg discs, while Dlp expression was effectively inhibited in *en^ts^* > *dlp*-RNAi leg discs (Figure 1E). This indicates that the stocks we used are efficient to manipulate *dlp* expression levels. The statistical analysis showed that the posterior compartment of *en^ts^* > *dlp* leg discs was also significantly reduced (Figure 1F).

### 3.2. Excess Dlp Induces Morphology Modification of Leg Disc with No Apparent Cell Proliferation Alteration

**Figure 2:**
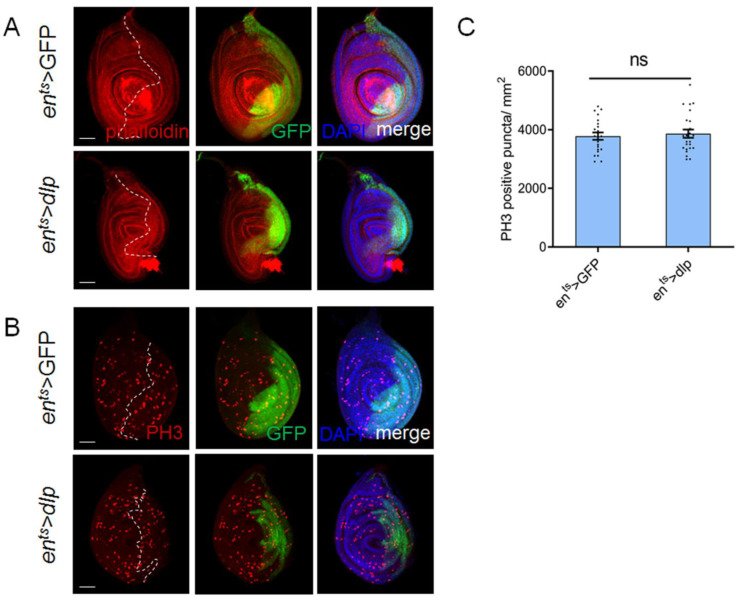
Excess Dlp induced shrinkage of leg discs with no apparent cell proliferation alteration. (**A**). Phalloidin staining reveals normal leg disc morphology in the control group (*en^ts^* > GFP), while overexpression of Dlp results in morphological deformities in the P compartment. (**B**). Staining with anti-PH3 reveals no apparent change in cell proliferation rate between the control and Dlp-overexpressing leg discs. (**C**). Statistical analysis of the PH3-positive puncta density (mean ± SEM; *en^ts^* > GFP, *n* = 22; *en^ts^* > *dlp*, *n* = 24). It reveals no apparent change in the cell proliferation rate between the control and Dlp-overexpressing leg discs. The white dashed lines indicate the A–P compartment boundary. ns means no statistically significant differences. The scale bar is 50 μm.

In consideration of the visible effects of overexpression rather than repression of Dlp, we focused our investigation on the *en^ts^* > *dlp* leg discs in the following study. Next, we utilized rhodamine–phalloidin staining to visualize F-actin within the leg discs, providing insights into the morphology of leg discs. In line with the observed deformities in the adult leg structure, the leg discs of *en^ts^* > *dlp* flies also exhibited abnormal shapes (Figure 2A). This correlation suggested that elevated Dlp expression leads to leg morphology modification even at early developmental stages.

The reduced P compartment might be caused by abnormal cell proliferation. Next, we assessed the cell proliferation rate using anti-PH3 staining. It revealed that there was no apparent alteration of cell proliferation in the P compartment of *en^ts^* > *dlp* discs; this was achieved by counting the PH3-positive puncta (Figure 2C). Considering that the shrunken P compartment might influence accurate quantification, we also examined leg discs expressing Dlp for 2 days, which exhibited a lesser reduction in the P compartment and fewer deformities (Figure S2A,B). Notably, this experiment also demonstrated no apparent alteration of cell proliferation in the P compartment (Figure S2C). These findings suggest that the leg deformities might not result from the cell proliferation alteration.

### 3.3. Excess Dlp Induces Apoptotic Cell Death

**Figure 3:**
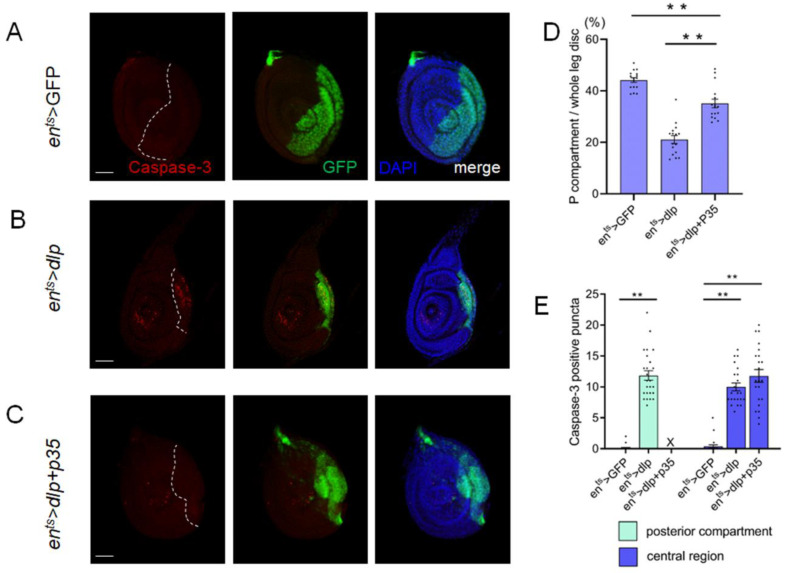
Dlp overexpression induces cell apoptosis both autonomously and non-autonomously. (**A**). No apparent cell death is detected in the control leg disc. (**B**). Overexpression of Dlp induces marked cell apoptosis within the P compartment (autonomous) and in regions outside of Gal4 expression (non-autonomous). (**C**). Expression of p35 blocks the cell apoptosis in the P compartment, while non-autonomous cell death still persists in the central region of leg disc. (**D**). The size of P compartment is partially rescued by co-expression of *p35* (mean ± SEM; *en^ts^* > GFP, *n* = 16; *en^ts^* > *dlp*, *n* = 16; *en^ts^* > *dlp* + *p35*, *n* = 16) (**E**). Cell death in P compartment is inhibited totally by *p35*, while the non-autonomous cell death in central region is still severe (mean ± SEM; *en^ts^* > GFP, *n* = 23; *en^ts^* > *dlp*, *n* = 25; *en^ts^* > *dlp* + *p35*, *n* = 16). The white dashed lines indicate the A–P compartment boundary. Asterisks indicate significant differences (*p* < 0.01). The scale bar is 50 μm.

Cell death plays a pivotal role in tissue homeostasis and organ development. Abnormal signaling frequently triggers cell apoptosis. We wonder whether the malformed leg morphology could be attributed to cell death elicited by the overexpression of Dlp. In control leg discs, no noticeable apoptosis was detected within the discs (Figure 3A). We observed apparent cell death in the posterior compartment of the *en^ts^* > *dlp* discs, as labeled by anti-Caspase-3 staining. Additionally, non-autonomous cell death also emerged in the anterior compartment (Figure 3B). This implied that the defect caused by excess Dlp expression may be mediated by cell death.

To further investigate whether the Dlp-induced leg deformities were caused by cell apoptosis, we co-expressed P35, a known cell death inhibitor, to block cell death. Caspase-3 detection showed that *p35* efficiently repressed cell death, while non-autonomous cell death was still present outside the Gal4 region (Figure 3C,E). Furthermore, it was observed that the size of the P compartment was partially rescued (Figure 3D). However, most of the *en^ts^* > *dlp* + *p35* flies failed to develop into the adult stage. Thus, we cannot directly assay the rescue effect.

### 3.4. Extra Dlp Induces Wg Mis-Distribution

**Figure 4:**
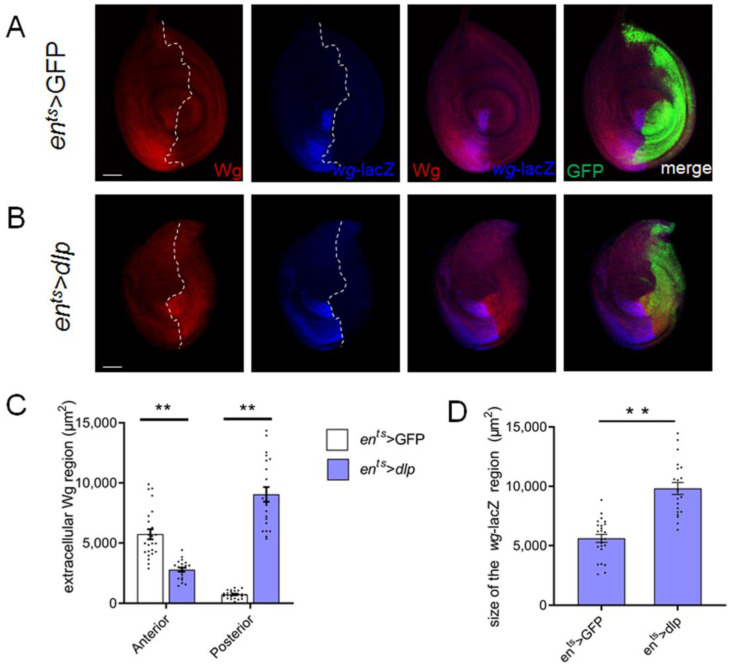
Excess Dlp changes Wg distribution in the leg disc. (**A**). In the control leg disc, the expression of *wg*-lacZ and the majority of Wg distribution are confined to a wedge-shaped region within the A compartment. (**B**). Overexpression of Dlp expands the Wg distribution domain within the P compartment, while concurrently causing a reduction in the distribution domain within the A compartment. *wg*-lacZ expression domain is enlarged. The white dashed lines indicate the A–P compartment boundary. (**C**). In the *en^ts^* > *dlp* leg disc, the Wg distribution domain in the P compartment is expanded; it is decreased in the A compartment (mean ± SEM; *en^ts^* > GFP, *n* = 24; *en^ts^* > *dlp*, *n* = 22). (**D**). The *wg*-lacZ expression domain is enlarged in the *en^ts^* > *dlp* leg disc (mean ± SEM; *en^ts^* > GFP, *n* = 24; *en^ts^* > *dlp*, *n* = 21). Asterisks indicate significant differences (*p* < 0.01). The scale bar is 50 μm.

Wg is typically expressed in the ventral half of the leg disc (Figure 4A) and plays a pivotal role in leg development. Aberrant Wg signaling is frequently linked to the malformation of organs [29,30]. Previous studies have indicated that cells mutant for Dlp disrupted the Wg distribution and affected Wg diffusion in wing discs [14]. Furthermore, it has been shown that Dlp exhibits contrasting effects at low and high levels of Wg, promoting low-level Wg activity while inhibiting high-level Wg activity. To investigate whether the leg deformities were due to mis-regulated Wg signaling, we performed double staining with the Wg antibody and *wg*-lacZ in the leg disc. In line with a previous study [27], Wg is predominantly distributed in the anterior compartment and only has a small distribution region in the P compartment in the normal leg disc, and *wg*-lacZ was also restricted in a wedge-shaped region in the anterior compartment, roughly overlapping with the distribution area of Wg (Figure 4A). Intriguingly, excess Dlp expanded the posterior distribution domain of Wg, accompanied by a reduction in the anterior Wg distribution region (Figure 4B,C). Our findings also revealed that *wg*-lacZ expression domains were notably expanded (Figure 4B,D), which is likely regulated by a feedback loop mechanism. These observations collectively suggest that excess Dlp alters the spatial distribution of Wg in the leg disc.

### 3.5. Excess Dlp Causes Mis-Expression of Leg Patterning Gene Dll

**Figure 5:**
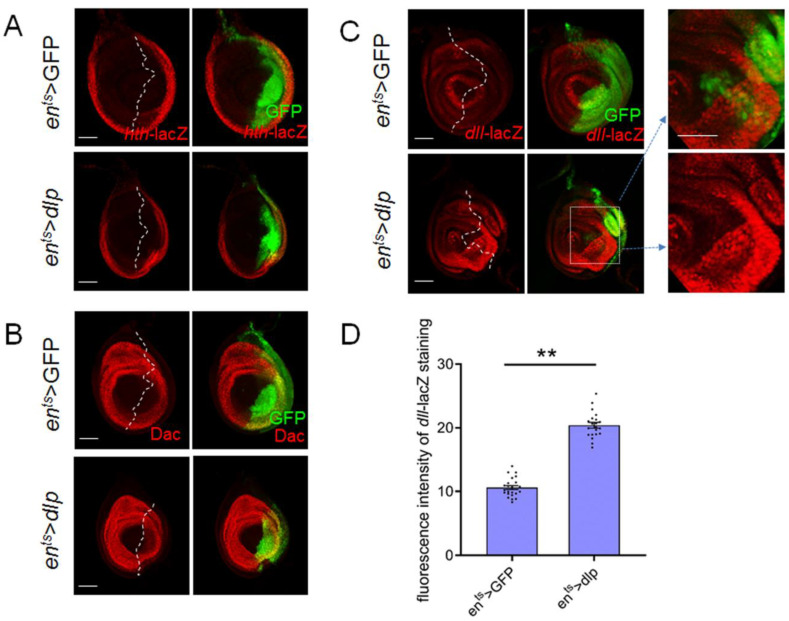
Excess Dlp causes mis-expression of *dll* but not notable alteration of *hth* and *dac*. (**A**). Excess Dlp has no apparent effect on the *hth* expression pattern. (**B**). Excess Dlp has no apparent effect on the Dac expression pattern. (**C**). Excess Dlp causes up-regulated *dll* in the edge region of the P compartment and the region adjacent to the *en*-Gal4 domain. (**D**). The statistical analysis shows that the *dll* expression is elevated significantly (mean ± SEM; *en^ts^* > GFP, *n* = 23; *en^ts^* > *dlp*, *n* = 21). The white dashed lines indicate the A–P compartment boundary. Asterisks indicate significant differences (*p* < 0.01). The scale bar is 50 μm.

*Drosophila* legs consist of multiple segments, including the coxa, trochanter, femur, tibia and tarsa along the P–D axis. The leg segmentation is controlled by some key regulators, including *hth*, *dac* and *dll*. *dll* is expressed in the central domain of the leg disc and directs the patterning of the future distal tip of the leg, whereas *dac* is expressed in an intermediate ring in the leg disc where it partially overlaps with the *dll* domain. It gives rise to the presumptive more proximal leg structures such as the femur and tibia. Additionally, *hth* is expressed in a peripheral ring of the leg disc, which corresponds to the most proximal part of adult legs. As mentioned above, *wg* signaling positively regulates *dll* and *dac* expression [31]. Next, we detected whether the excess Dlp affected the expression pattern of these genes by employing a lacZ reporter or antibody to monitor the expression of these factors. Our results revealed that excess Dlp led to the up-regulation of *dll* expression in the posterior region. Furthermore, non-autonomous up-regulation of *dll* expression was observed in the ventral domain adjacent to the *en*-Gal4 region (Figure 5C). To confirm this result, we also detected different focal planes of the leg disc and observed an evident elevation of *dll* expression (Figure S3). Additionally, the expression patterns of *dac* and *hth* in this context appear relatively normal (Figure 5A,B). A previous study has demonstrated that *dll* overexpression in the leg disc induces abbreviated and fused distal segments of adult legs [32], resembling the adult leg phenotype observed in our study. Thus, we deduce that the observed leg malformations might be related to abnormal *dll* expression induced by excess Dlp, potentially associated with alterations in the Wg distribution.

## 4. Discussion

HSPGs are macromolecules widely distributed in the extracellular matrix of cells. They play a crucial role in various biological processes, including the maintenance of tissue homeostasis and involvement in tumorigenesis [10]. HSPGs have been implicated in regulating the growth and patterning of numerous tissues and organ systems. Mis-expression of these molecules may lead to deformities in developing organs [8]. On the other hand, the mechanisms underlying organ development and patterning in both chordate and arthropod phyla share numerous similarities. *Drosophila* legs provide an excellent model to investigate these mechanisms governing organ formation. In this study, we observed that overexpression of Dlp, one of the *Drosophila* HSPGs in the posterior compartment of leg discs under the control of the *en*-Gal4 driver, resulted in the malformation of adult legs (Figure 1A,B). Similar results were obtained with the anterior Gal4 driver, *dpp*-Gal4. Considering the relatively large domain of *en*-Gal4, we focused on manipulating Dlp expression specifically in the P compartment using *en*-Gal4.

### 4.1. Cell Death Is the Potential Factor Inducing Deformities of Leg Discs

The reduction in the P compartment size may be caused by the activation of cell apoptosis or hindered cell proliferation. Disordered cell signaling frequently leads to cell death in various developing tissues. Our study revealed that excess Dlp induced a significant increase in cell death both autonomously and non-autonomously (Figure 3A,B). Why does the excess Dlp induce cell death in the center of leg discs non-autonomously? Based on some studies, apoptotic cells in one compartment of the Drosophila imaginal disc release long-range death factors such as Eiger, which induces apoptosis in an adjacent compartment [33]. This could be a potential reason. Alternatively, some reports have indicated that a reduction in Wg levels also promotes cell apoptosis [34]. It is plausible that an abnormal distribution of Wg triggered by excess Dlp reduces the Wg signals received by cells in the central region of the leg disc. Alternatively, cells situated in the center might display increased sensitivity to changes in Wg signaling, making them more prone to apoptosis when Wg levels are reduced. Furthermore, co-expression of p35 partially rescued the reduction in the posterior compartment. Therefore, we hypothesize that cell death plays an important role in the induction of leg deformities.

### 4.2. Excess Dlp Results in Abnormal Distribution of Wg

Wingless (Wg) is a crucial morphogen guiding organ patterning [29,30]. Previous studies have suggested that Dlp is necessary for Wg movement and gradient formation in the wing disc. Additionally, Dlp is considered a co-factor in Wg signaling. It has been demonstrated that Dlp exerts opposing effects on Wg activity at the domains with low and high levels of Wg. In cells with low levels of Wingless activity, Dlp acts as a positive co-factor to enhance Wg activity. Conversely, Dlp reduces Wg activity in cells with high levels of Wingless activity [17]. A previous study demonstrated that aside from the Wg source region in the A compartment, Wg is also distributed in a small area within the P compartment [28]. In addition, it has been shown that overexpression of Dlp results in an accumulation of extracellular Wg in the wing disc [35]. Our findings indicate that an excess of Dlp leads to the expansion of Wg distribution within the P compartment. Conversely, the region of Wg diffusion within the A compartment is notably reduced (Figure 4A,B,D). This implies that cells with higher Dlp recruit soluble Wg competitively. Dlp consists of a core protein with several attached HS GAG chains. It has been shown that the Dlp core protein exhibits biphasic activity similar to that of wild-type Dlp. The attached GAG chains appear to enhance the interaction between the Dlp core protein and Wg [36]. Therefore, we posit that excess Dlp provokes alterations in Wg distribution primarily through its core protein. Moreover, non-autonomously, the domain of *wg*-lacZ in its source region is enlarged noticeably. This suggests the existence of an unknown mechanism regulating *wg*-lacZ expression, potentially involving a feedback loop.

### 4.3. Compared to Dac and Hth, Dll Is More Responsive to the Excess Dlp

*Drosophila* legs consist of several segments along the proximal–distal (P–D) axis. In our study, conspicuous deformities were consistently observed in the distal part of adult legs. Interestingly, the repression of *dlp* exhibited minimal indications of abnormal leg patterning (Figure 1A–D). The orchestration of distinct signaling pathways, including *hth, dac* and *dll*, were identified as pivotal in mediating the development of different leg segments [37]. Specifically, *hth* has been shown to govern the proximal region [26,38], *dac* the medial region [28] and *dll* the distal part of legs [37]. Previous research has highlighted the involvement of Wg signaling in activating the expression of Dac and Dll [28], whose expressions display varying sensitivities to Wg signaling levels [39]. Our findings demonstrated an elevated *dll* expression in leg discs, while *dac* and *hth* exhibited no significant modifications (Figure 5). It has been shown that overexpression of *dll* in leg discs caused abbreviated and fused distal segments of legs [26], which is very similar to the legs observed in our study, providing support for this conclusion. Consequently, we suggest that the impact of excess Dlp on leg development involves the modulation of key patterning factors of legs.

### 4.4. The Limitations of the Study

It is essential to acknowledge the limitations of the study, such as the specific focus on Dlp and the need for further exploration of the other glypican *dally*. Additionally, our study revealed that the *wg*-lacZ domain in its source region was enlarged non-autonomously (Figure 4A,B,D). The regulation mechanism remains elusive. Additionally, our study revealed missing and mis-localization of legs, suggesting a potential implication of HSPGs in organ positioning. Abnormal Wnt signaling can lead to a variety of limb defects, including missing limbs or Ectrodactyly-hand and split-foot malformations [40,41]. In mice, mutation of glypican-3 leads to defects such as Polydactyly [42]. The observation of missing or mis-localized legs could serve as a valuable model for studying the etiology of finger and arm misalignment. Subsequent research endeavors could delve into the underlying molecular mechanisms and explore potential interactions with other regulatory factors. This knowledge may extend beyond *Drosophila*, offering valuable insights into the broader field of developmental biology.

## 5. Conclusions

**Figure 6:**
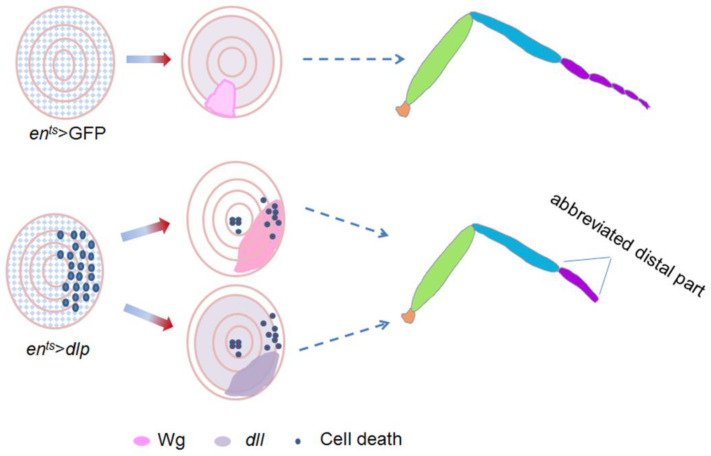
Schematic diagram illustrating the effects of excess Dlp in the P compartment of the leg disc. It leads to abnormal Wg distribution, up-regulated *dll* expression and noticeable cell death, which potentially contribute to the abbreviated distal part of adult legs.

This study sheds light on the important role of Dlp signaling in regulating *Drosophila* leg development. We demonstrate that excess Dlp induces a notable shrinkage of the adult leg distal part, accompanied by evident cell death but no apparent alterations in cell proliferation. Furthermore, our findings reveal an impact on Wg distribution and elevated Dll expression (Figure 6). Given that Dll directly guides the patterning of the leg’s distal part and Wg tightly regulates Dll activity, we propose that the alterations of these two signals, as well as the induced cell death, are the potential factors responsible for the observed leg malformations. Our findings enrich our comprehension of the regulatory networks underlying *Drosophila* leg development and facilitate future exploration into the complex molecular mechanisms controlling organ pattern formation.

## Data Availability

All the data generated in this work were provided in the article.

## Supplementary Materials

The following supporting information can be downloaded at https://www.mdpi.com/article/10.3390/cells13141199/s1, Figure S1. *dlp* knockdown flies (*en^ts^* > *dlp*-RNAi) exhibit normal leg location, whereas flies overexpressing *dlp* (*en^ts^* > *dlp*) display a phenotype characterized by missing and mis-located legs compared to the control group (*en^ts^* > GFP). Red circles highlight the basal locations of the legs in the flies. The scale bar is 0.5 mm. Figure S2. Overexpressing Dlp in the P compartment for 2 days. A. PH3 staining of *en^ts^* > GFP leg discs. B. PH3 staining of *en^ts^* > *dlp* leg discs activated at 30 °C for 2 days. C. The ratio of the number of posterior PH3-positive puncta to the area size of the P compartment (mean ± SEM; *en^t^ ^s^*> GFP, *n* = 20; *en^ts^* > *dlp*, *n* = 17). ns means no statistically significant differences. The scale bar is 50 μm. Figure S3. Different focal planes depicted in the *dll*-lacZ staining in the bottom panel of Figure 5C demonstrate an up-regulation of *dll*-lacZ.
