# Supplementary figures and images for: Excess Dally-like Induces Malformation of Drosophila Legs
Source: Cells. 2024 Jul 15;13(14):1199. doi: 10.3390/cells13141199 (PMC11274743; doi:10.3390/cells13141199)

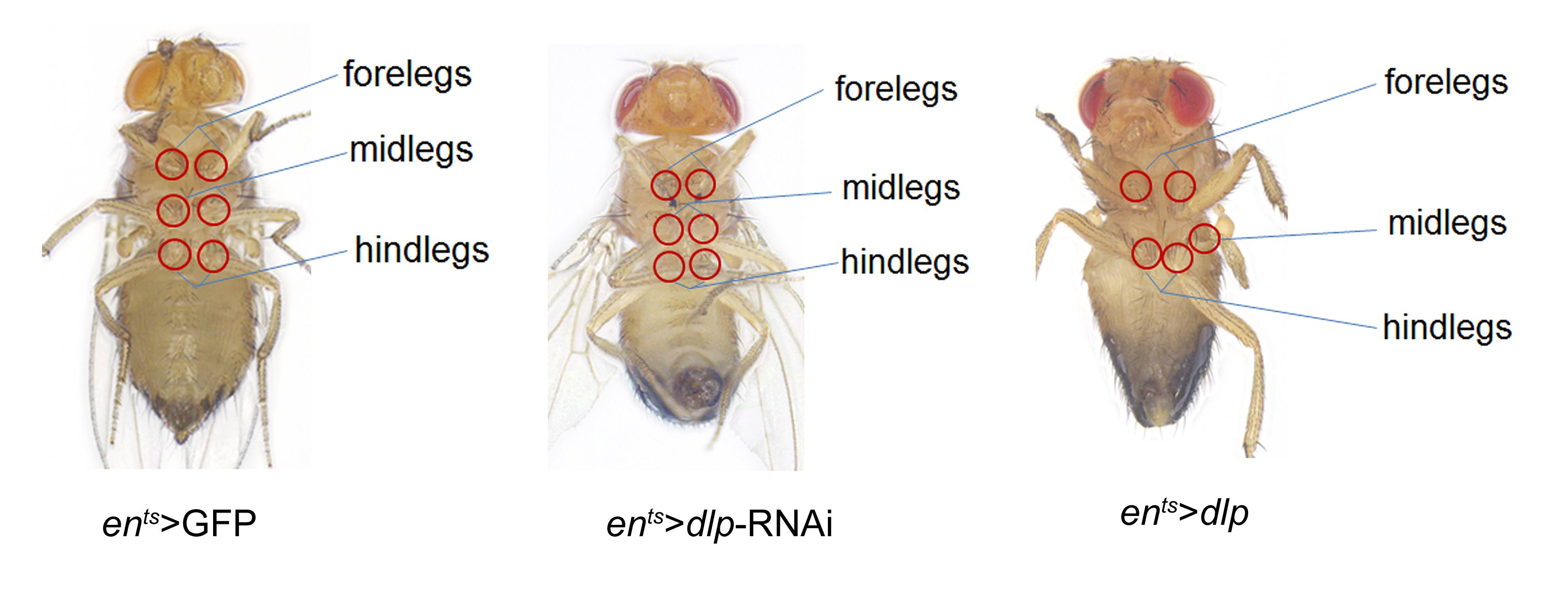

Supplement: Supplementary file 1 [file cells-13-01199-s001.zip › Supplementary File/Fig S1.tif]

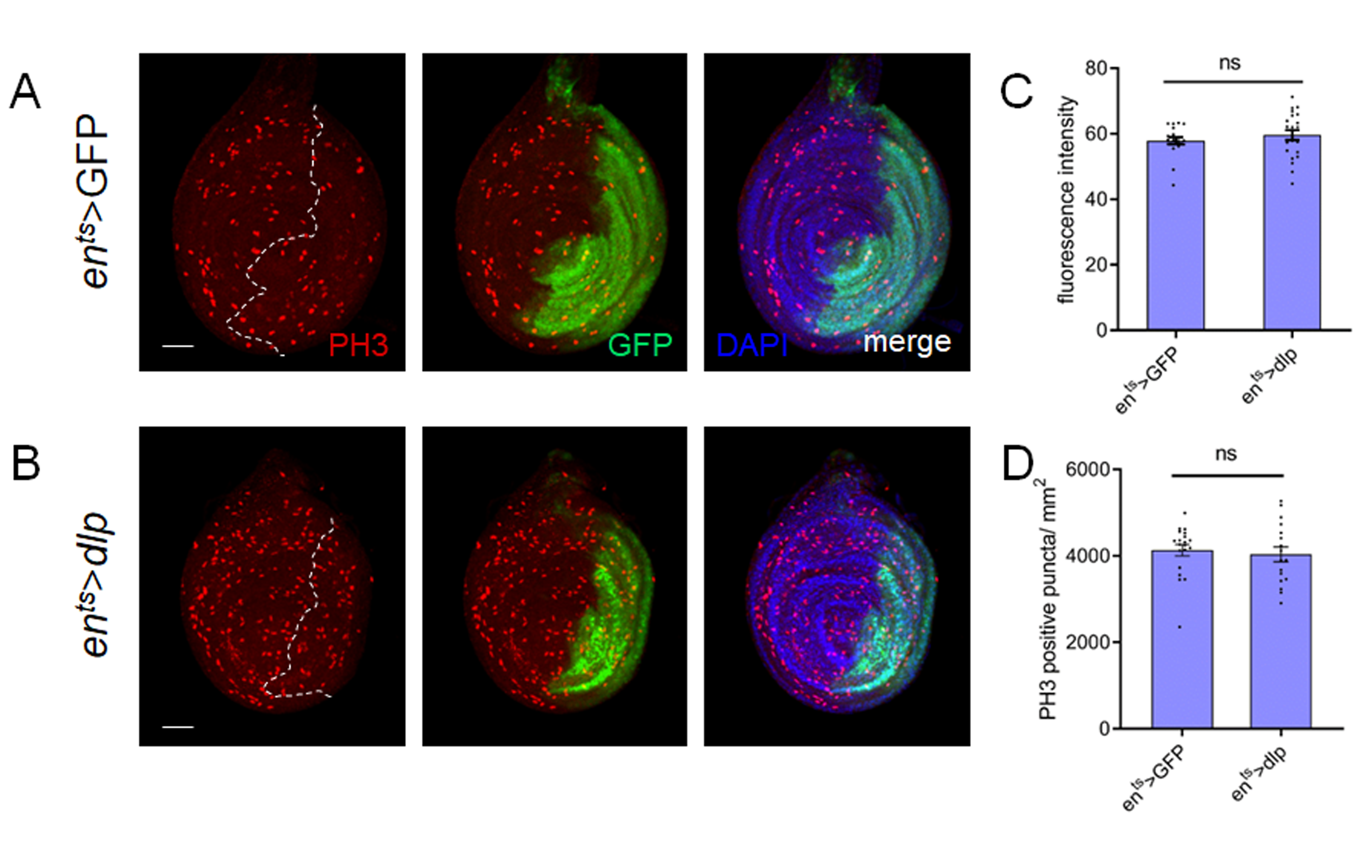

Supplement: Supplementary file 1 [file cells-13-01199-s001.zip › Supplementary File/Fig S2.tif]

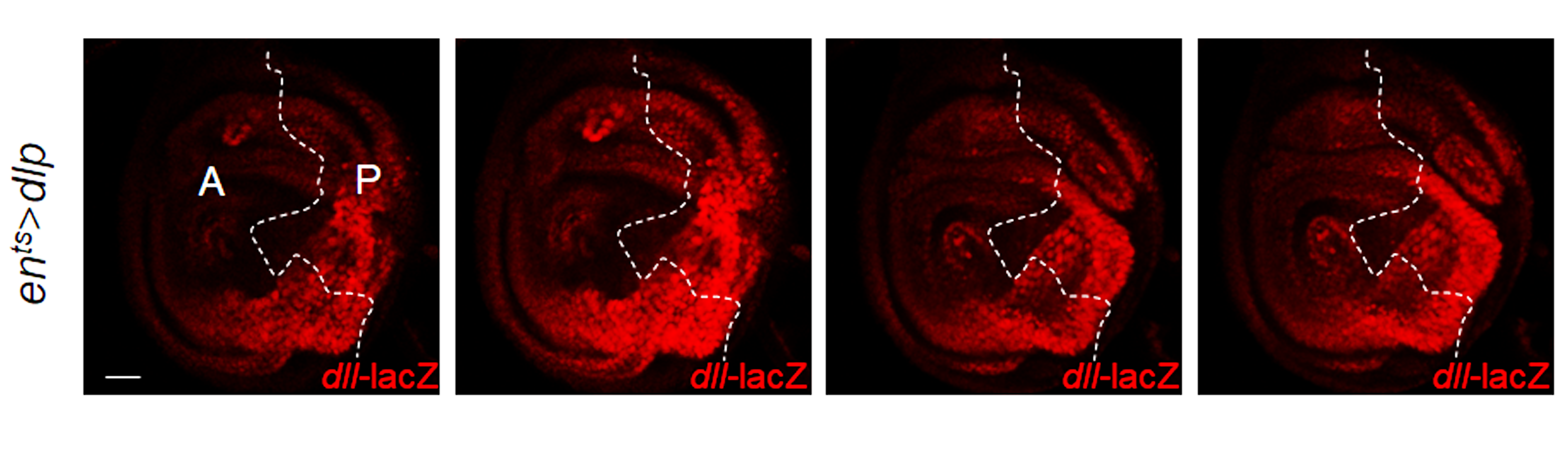

Supplement: Supplementary file 1 [file cells-13-01199-s001.zip › Supplementary File/Fig S3.tif]
